# The Need for Head Protection Protocols for Craniectomy Patients during Rest, Transfers and Turning

**DOI:** 10.3389/fsurg.2022.918886

**Published:** 2022-05-20

**Authors:** Anand S. Pandit, Prabhav Singhal, Sogha Khawari, Astri M. V. Luoma, Sara Ajina, Ahmed K. Toma

**Affiliations:** ^1^ Department of Neurosurgery, National Hospital for Neurology and Neurosurgery (NHNN), London, United Kingdom; ^2^ UCL Medical School, Faculty of Medicine, UCL, London, United Kingdom; ^3^ Department of Neuroanaesthesia and Neurocritical Care, NHNN, London, United Kingdom; ^4^ Department of Neuro-rehabilitation, NHNN, London, United Kingdom

**Keywords:** head protection, decompressive craniectomy, TBI, malignant stroke, patient transfer

## Abstract

After craniectomy, patients are generally advised to wear a helmet when mobilising to protect the unshielded brain from damage. However, there exists limited guidance regarding head protection for patients at rest and when being transferred or turned. Here, we emphasise the need for such protocols and utilise evidence from several sources to affirm our viewpoint. A literature search was first performed using MEDLINE and EMBASE, looking for published material relating to head protection for patients post-craniectomy during rest, transfer or turning. No articles were identified using a wide-ranging search strategy. Next, we surveyed and interviewed staff and patients from our neurosurgical centre to ascertain how often their craniectomy site was exposed to external pressure and the precautions taken to prevent this. 59% of patients admitted resting in contact with the craniectomy site, in agreement with the observations of 67% of staff. In 63% of these patients, this occurred on a daily basis and for some, was associated with symptoms suggestive of raised intracranial pressure. 44% of staff did not use a method to prevent craniectomy site contact while 65% utilised no additional precautions during transfer or turning. 63% of patients received no information about avoiding craniectomy site contact upon discharge, and almost all surveyed wished for resting head protection if it were available. We argue that pragmatic guidelines are needed and that our results support this perspective. As such, we offer a simple, practical protocol which can be adopted and iteratively improved as further evidence becomes available in this area.

## Introduction

Decompressive craniectomy remains a mainstay of treatment for patients with refractory elevated intracranial pressure, including patients with intracerebral haemorrhage and malignant stroke. Patients are typically advised to wear helmets after the craniectomy to avoid injury to the unprotected brain when mobilising (1). In contrast, the utility of head protection in supine or semi-recumbent positions at rest is unclear. Although patients are less likely to experience high-impact injury when recumbent, prolonged periods of pressure when patients lie in contact with their surgical site may result in repetitive microtrauma to the unshielded brain (Supplementary Figure S1). Separately, low to moderate-impact injury may occur when the patient is transferred or turned without additional precautions to keep the craniectomy site secure.

These issues are exacerbated threefold for decompressive craniectomy patients in critical care: (i) these patients may be within the window for acute management of raised intracranial pressure (ICP) and would be especially vulnerable to any exogenous insult that may increase their ICP; (ii) many patients are paralysed and lack the ability to maintain a neutral head position; and (iii) patients are often unconscious and unable to communicate if external pressure is applied. While patients outside of critical care may also be exposed to some of the aforementioned conditions, it is probable that individuals in the acute window of treatment have the greatest risk of injury and may benefit the most from head protection.

In this perspectives article, we emphasise the need for head protection protocols for patients at rest and when being turned or transferred. We utilise evidence from several sources to affirm our viewpoint. Firstly, a scoping literature review was performed to confirm whether published evidence existed regarding the use of head protection for craniectomy patients at rest, during transfers and turning. Secondly, we determined from staff in critical care and ward-based settings within our own institution, the frequency of contact between craniectomy site and external surfaces and whether advice or protocols were available that would help prevent this occurring. Thirdly, in patients who had a decompressive craniectomy and could communicate, we wanted to investigate patients’ experiences regarding resting pressure on their craniectomy site and their opinions concerning head protection. Finally, we offer our anecdotal experience regarding these issues.

## Materials and Methods

### Ethics

This study was approved by our institutional review board as a service evaluation (123-202021- CA) and informed consent was gained from all interviewed staff and patients.

### Systematic Review

A systematic search was performed for articles from electronic databases including MEDLINE and EMBASE from database inception until 23/12/21. Our search terms and strategy are outlined in Supplementary Table S1 and Figure S2 respectively.

The following criteria were defined for inclusion: English-only abstracts for patients of any age and any of the following types of manuscript: case reports, case-control studies, systematic reviews and meta-analyses, randomised control trials, letters to the editor and observational studies. Articles where the abstracts were published in a language other than English were excluded.

### Patient and Staff Interviews

Using the electronic healthcare record from our centre (a large tertiary, academic neurosciences unit), we identified adult patients who had a craniectomy performed since the record’s inception (31 March 2019) to 31 July 2021. Patients with a posterior fossa craniectomy (PFC) were excluded since the resting interface pressure for these patients when sleeping supine is typically over the occiput rather than the posterior craniectomy site, which is located more inferiorly (2).

Using the above criteria, we identified 100 patients (34F/66M with mean age 50.7 years [SD 12.5]) that had a craniectomy during that period. We then sought which of these patients would be suitable participants for semi-structured interviews i.e., could communicate verbally, or in a way which could be understood over telephone, have a Glasgow Coma Score (GCS) of 15, had no cognitive deficits related to their memory or recall: allowing them to make an informed consent for the study.

33 patients were contacted between 22 March 2021 to 29 August 2021 (Table 1). From the 67 patients that did not meet our interview criteria, 18 patients were deceased, 37 patients had a GCS of less than 15 or substantial neurocognitive impairments, and 12 patients had significant aphasia. 33 patients met our criteria for interviews of which 27 were completed (82% response rate). Patients commented upon their frequency of craniectomy site contact, any symptoms experienced (Supplementary Table 2) and how well informed they were regarding craniectomy protection (Table 1).

**Table 1:** Patients and staff responses regarding craniectomy site contact, its consequences and use of devices to mitigate this risk.

Patients						
Characteristic	Result					
*n*	27					
Mean age ±SD (yrs)	47.3±11.9					
Female	7 (26%)					
Indication for craniectomy	Neurovascular	17 (63.0%)	Malignant stroke	13 (48.1%)		
			Intracerebral haemorrhage	2 (7.4%)		
			Subarachnoid haemorrhage	1 (3.7%)		
			Arteriovenous malformation	1 (3.7%)		
	Other	10 (37.0%)	Brain abscess	3 (11.1%)		
			Subdural haematoma	3 (11.1%)		
			Traumatic Brain Injury	2 (7%)		
			Encephalitis	1 (3.7%)		
			Meningioma	1 (3.7%)		
Recall sleeping on same side and in contact with craniectomy site	Yes	16 (59.3%)	*Frequency*		*Symptoms*	
			Daily	10/16 (62.5%)	Local	7/16 (43.8%)
			More than once a week	1/16 (6.3%)	Systemic	2/16 (12.5%)
			Once a week	2 /16 (12.5%)	None	7/16 (43.8%)
			Unsure	3/16 (18.8%)	Unsure	2/16 (12.5%)
	No	7 (3.7%)				
	Unsure	4 (14.8%)				
Told to avoid pressure over craniectomy site prior to discharge	Yes	10 (37.0%)				
	No	12 (44.4%)				
	Unsure	5 (18.5%)				
Want a device to prevent contact with craniectomy site when resting	Yes	24 (88.9%)				
	No	1 (3.7%)				
	Unsure	2 (7.4%)				
**Staff**						
*N*	48					
Role	Nurse	32 (66.7%)				
	Doctor	10 (20.8%)				
	Care Assistant	2 (4.2%)				
	Physiotherapist	4 (8.3%)				
Ward type	Neurology/Stroke	10 (20.8%)				
	Neurosurgery	7 (14.6%)				
	Neuro-rehabilitation	13 (27.1%)				
	Neuro ICU	18 (37.5%)				
Median no. of unique craniectomy patients cared for in 2 week audit period	2 [1–7]					
Observed external contact on craniectomy site	Yes	32 (66.7%)	*Median number observed [range]*	1 [1–4]	*Surface*	
					Pillow	12/32 (37.5%)
			*** ***		Bedside	2/32 (6.3%)
			*** ***		No response	18/32 (56.3%)
	No	14 (29.2%)	*** ***			
	Unsure	2 (4.2%)	*** ***			
Use a device for keeping head neutral at rest	Yes	27 (56.3%)	*Device*			
			Pillows	16/27 (59.3%)		
			Towels	16/27 (59.3%)		
			Bed positioning	1/27 (3.7%)		
	No	21 (43.8%)				
Use additional precautions for craniectomy site protection during patient transfers/turning	Yes	17 (35.4%)	*Method*			
			Use helmet	9/17 (53.0%)		
			Additional staff for head protection	2/17 (11.8%)		
			Hoist	2/17 (11.8%)		
			Not-specified	4/17 (23.5%)		
	No	31 (64.6%)	*** ***			

In an overlapping two-week period, between 18 April 2021 to 4 May 2021, staff members of all disciplines were surveyed, including medical, surgical, nursing, and auxiliary therapies; all actively involved in the care of craniectomy. Responses from 48 staff members, working across intensive care, neurology, neurosurgery and neuro-rehabilitation wards were obtained. Staff commented upon the frequency of craniectomy site contact with an external surface and how this was mitigated against at rest and during patient transfer and turning (Table 1).

## Results and Discussion

Decompressive craniectomy as a treatment for refractory intracranial hypertension following malignant stroke and traumatic brain injury is anticipated to increase (3–5). Craniectomy patients typically spend a considerable period of time waiting for a cranioplasty (6), during which, the brain is unshielded and vulnerable to mechanical injury (7). Whereas helmets are typically offered to patients when mobilising, guidance regarding head-protection at rest, when turning and transferring is unclear.

In the absence of established guidelines, we advocate for a simple, common-sense protocol for craniectomy patients in these circumstances (Table 2), significantly extending the instructions offered by Livesay and Moser (1). In our recommendations, we call for greater awareness of the condition of these patients among staff (items 1 and 2), emphasise the need to maintain neutral head positioning (item 3), take precautions on transfers, turning and mobilising (item 5) and offer clear advice to the patient and their home care team (item 6).

**Table 2:** Suggested standard operating protocol for post-operative care of craniectomy patients.

Item	Recommendation	Setting
1	Once a patient is transferred from theatre, ensure the craniectomy site is clearly marked (e.g., with an adhesive label) and a sign placed at the bedside	ICU, ward
2	In written documentation and on handovers, ensure that receiving staff are aware the patient has had a craniectomy	ICU, ward
3	If a patient is seen resting with their craniectomy site in contact with an external surface, reposition the head to a safe position, if necessary, with rolled towels, pillows or a soft collar (if not contraindicated)	ICU, ward
4	When transferring or turning the patient, ensure that at least one staff member is supporting the head and protecting the craniectomy site	ICU, ward
5	Once a helmet is available, ensure it is always worn when mobilising and mark patient as a high falls risk	Ward
6	Upon discharge, provide the patient or their carers with a written copy of the above instructions and that contact with the craniectomy site should be avoided.	Ward

*ICU, intensive care unit.*

Our viewpoint and protocol are supported by several lines of argument based on our scoping review, ancillary evidence and our own institutional experience via patient and staff surveys. We first systematically demonstrate a lack of evidence in this area, and the rationale for developing a protocol. Our comprehensive search strategy identified no articles that discussed head protection or risk of injury, post-craniectomy for patients at rest or when being transferred or turned. While it may not be surprising that original research is lacking in this domain, we would argue that this lack of clarity is detrimental for both staff and patients. In the absence of established guidance, staff are likely to be in doubt regarding craniectomy patient care including which nursing and supportive measures to implement and appropriate advice to offer patients in hospital and upon discharge. Patients also may be in doubt about which resting measures to adopt in order to protect their unshielded brain, contributing to the pre-existing anxiety levels in this population (8) and potentially risking further harm (see below). These uncertainties are also reflected in the responses from our institutional survey.

We found from both staff and craniectomy patients who could communicate, that external contact with their craniectomy site occurs regularly. 59% of patients stated they slept on the same side as their craniectomy site, with the majority indicating that this occurred on a daily basis (Table 1). This was in agreement with 67% of multi-disciplinary staff, who had witnessed that patients rest with their craniectomy site directly in contact with pressure from an external surface.

We also observed that staff are poorly informed whether to maintain a neutral head-position at rest or during patient transfer and turning and the means to achieve this. Approximately half of the interviewed staff did not use any method to maintain a neutral resting head position to avoid contact with the craniectomy site, while the remainder utilised pillows, towels or bed-positioning to do so. 65% of staff used no additional precautions to protect the craniectomy site during patient transfer or turning, such as use of a helmet, hoist or additional staff members. Equally, patients were poorly advised and did not receive craniectomy-specific care instructions upon discharge. Indeed, 63% of patients received no information or were unsure about avoiding contact over the craniectomy site following discharge (Table 1).

Resting craniectomy site contact among patients was associated with both local and systemic symptoms (Supplementary Table S2), some of which were suggestive of raised intracranial pressure (9). More than a quarter of interviewed patients complained of systemic symptoms such as headache, dizziness and nausea and/or local sensations including pain and paraesthesia (Supplementary Table S2). It is possible that other confounding factors may have contributed to the patients’ symptoms such as syndrome of the trephined (5), however none in our cohort had this as an explicit diagnosis at the time of survey in their hospital record.

We acknowledge that the true risk of neurological harm in this situation is, as yet, unquantified and some may argue that if a patient’s craniectomy site is in contact with a soft pillow, with ample time for the brain volume to adjust, the chance of significant brain injury would be low. However, in rebuttal, we highlight that the resting interface of the mattress and gravity, can exert approximately 8 kPa (10) or 60 mmHg of external pressure upon the head. We posit that in patients who are sensitive to increases in ICP, particularly those within the acute window of ICP management following a traumatic brain injury or stroke, such an event would risk significant neurological injury.

Anecdotally within our unit, we have witnessed several patients who inadvertently rested with their craniectomy site in contact with an external soft surface, for potentially prolonged periods. Of this group, in those who had ICP monitors in-situ, pathological ICP levels were observed before returning to normal levels once their position was corrected. Certainly, future research studies should aim to investigate the neurological sequelae of external pressure on the unprotected brain. In the acute setting, ICP monitoring would be a valuable outcome measure that is already widely used for these patients (11, 12). Correlation of ICP at the time of observed craniectomy site contact in a prospective study would provide a truer estimation of transmitted interface pressure. For chronic patients, the long-term impact may be investigated through longitudinal neuroimaging. Here we might specifically observe for radiological markers of contusions or micro-trauma over the exposed cortical surface, some of which more easily seen in using susceptibility-weighting or advanced MRI sequences (13, 14).

Of key interest, despite the variable clinical sequelae following static pressure over the craniectomy site, nearly all the patients interviewed wished for resting head protection (Table 2) aimed at preventing surgical site contact if it were available. This may be in the form of appropriately sited pillows as described above, a soft collar or other positioning devices which would help in maintaining the head in a neutral position at rest.

Although, to the best of our knowledge, this is the first study and perspective article evaluating this issue, we acknowledge some limitations in the data used to support our viewpoint. Firstly, no objective measures (e.g., intracranial pressure recordings or neuroimaging) were used to demonstrate the effects of external pressure or if prolonged pressure affects long-term neurological outcomes. Here we speculate the risk of injury based on subjective patient experiences and ancillary evidence. Secondly, our findings only represent data from one centre and there may be differences in operating protocols and adherence when compared against other U.K. and international centres.

In spite of these limitations, however, our results demonstrate a clear interim need for pragmatic instructions in this area, in the absence of evidence-based guidelines. To help confirm or refute this protocol, further scientific enquiry is warranted. Research may also influence surgical decision-making, including cranioplasty timing. Recent systematic-level evidence suggests that performing a cranioplasty earlier (typically before 90 days) is associated with enhanced neurological function (6) and is likely to better restore cerebral homeostasis (15) without additional risk of complications (16). If the arguments outlined in this article are valid, they would add further support toward earlier timing of cranioplasty, here as a means of providing comprehensive and lasting brain protection for the patient.

## Data Availability

The datasets presented in this article are not readily available because of the ethical permissions and confidentiality regarding this information. Requests to access the datasets should be directed to a.pandit@nhs.net.
