# Supplementary material for: The Need for Head Protection Protocols for Craniectomy Patients during Rest, Transfers and Turning
Source: Front Surg. 2022 May 20;9:918886. doi: 10.3389/fsurg.2022.918886 (PMC9172832; doi:10.3389/fsurg.2022.918886)
Supplement: Supplementary file 1 [file Table_1_v1.docx]

**Supplementary Material: The Need for Head Protection Protocols for Craniectomy Patients during Rest, Transfers and Turning**

**Supplementary Methods**

**Supplementary Table 1. Systematic search terms**. Ovid Medline(R) ALL (1946 to 23 December 2021) and Embase (1974 to 23 December 2021). All fields searched with no limits = .mp. Date of repeat search: 24 December, 2021

|  | **Search** | **Results** |
| --- | --- | --- |
| **1** | (Craniect*).mp | 17831 |
| **2** | (Sleep* OR rest* OR supine OR lying OR lay* OR lie* OR sit* OR sat OR recumbent).mp | 9836924 |
| **3** | (Protect* OR support* OR safety).mp | 16184357 |
| **4** | (Pillow* OR cushion* OR helmet* OR collar* OR towel* OR headrest OR head ADJ rest OR immobil*).mp | 404979 |
| **5** | (Head OR skull OR cranium OR brain).mp | 4696524 |
| **6** | (Transfer* OR turn* OR therap* OR transport*) | 19611497 |
| **7** | 1 AND 3 | 2464 |
| **8** | 5 AND 7 | 2032 |
| **9** | 6 AND 7 | 1218 |
| **10** | 2 AND 7 | 471 |
| **11** | 1 AND 4 | 297 |
| **12** | 2 AND 11 | 54 |
| **13** | 4 AND 10 | 9 |
| **14** | 5 AND 13 | 8 |

**Supplementary Figure 1. Schematic diagram demonstrating the potential effect of sleeping on the same side as the craniectomy site.**

1. **Superior View B) Lateral View**

**
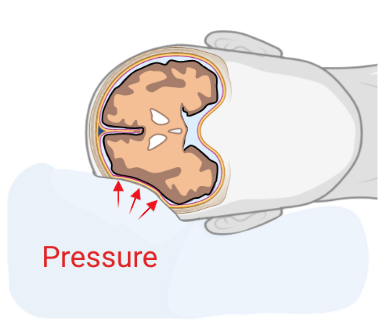

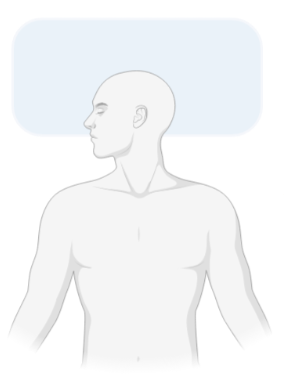
**

**Supplementary Figure 2. Systematic search strategy outlined as PRISMA flowchart**

Reports identified from:

1. Ovid Medline(R) ALL (1946 – 23rd December, 2021)
2. Embase (1974 – 23^rd^ December, 2021)

**Identification**

Reports (abstract) screened

(n = 19958)

Reports excluded:

1^st^ Reason: repeated studies (n= 652)

2^nd^ Reason: not written in English (n= 381)

3^rd^ Reason: study in paediatric population (n = 6954)

4^th^ Reason: study regarding helmet use for specific head shape or on helmet material (n = 8006)

**Screening**

Reports (full-text) assessed for eligibility (n = 3965)

Reports excluded:

1^st^ Reason: head protection only during mobilisation (n = 1985)

2^nd^ Reason: posterior fossa craniectomy (n = 321).

3^rd^ Reason: head position only during anaesthesia (n = 1659)

Studies identified which discuss head protection after craniectomy when patients are at rest/not mobilising or during patient transfer/turning (n = 0)

**Conclusion**

**Supplementary Results**

**Supplementary Table 2.** Patient subjective experiences after resting on same side and in contact with craniectomy site. (* describe local symptoms only, ** describe both local and systemic symptoms)

| **Patient ID** | **Symptoms** |
| --- | --- |
| 1 | “It’s a weird sensation, that is hard to describe.  It is like a suction feeling when I put my head on that side.” * |
| 2 | “After waking, I feel a bit dizzy, sick and have pain around the craniectomy site.” ** |
| 3 | “I get a jelly like sensation when I lay on that side.” * |
| 4 | “I wake up with pain in that area and often have a widespread headache” ** |
| 5 | “It's a bit sore but that's about it” * |
| 6 | “I thought I could feel some fluid but the doctors later told me that it was my brain protruding out. I was speechless.” * |
| 7 | “It feels like jelly when I put my head on that side” * |

**Supplementary References**

[S1]: Page MJ, Moher D, Bossuyt PM, Boutron I, Hoffmann TC, Mulrow CD, Shamseer L, Tetzlaff JM, Akl EA, Brennan SE et al. PRISMA 2020 explanation and elaboration: updated guidance and exemplars for reporting systematic reviews. BMJ. 2021 Mar 29;372:n160. doi: 10.1136/bmj.n160

[S2]: Cumpston M, Li T, Page MJ, Chandler J, Welch VA, Higgins JP, Thomas J. Updated guidance for trusted systematic reviews: a new edition of the Cochrane Handbook for Systematic Reviews of Interventions. Cochrane Database Syst Rev. 2019 Oct 3;10:ED000142. doi: 10.1002/14651858.ED000142
